# Proportional vascularization along the fallopian tubes and ovarian fimbria: assessment by confocal microtomography

**DOI:** 10.1590/0100-3984.2019.0080

**Published:** 2020

**Authors:** Pedro Teixeira Castro, Osvaldo Luiz Aranda, Edson Marchiori, Luiz Felipe Bittencourt de Araújo, Haimon Diniz Lopes Alves, Ricardo Tadeu Lopes, Heron Werner, Edward Araujo Júnior

**Affiliations:** 1 Universidade Federal do Rio de Janeiro (UFRJ), Rio de Janeiro, RJ, Brazil.; 2 Clínica Diagnóstico por Imagem (CDPI), Rio de Janeiro, RJ, Brazil.; 3 Universidade de Vassouras, Vassouras, RJ, Brazil.; 4 Universidade Federal Fluminense (UFF), Niterói, RJ, Brazil.; 5 Universidade do Estado do Rio de Janeiro (UERJ), Rio de Janeiro, RJ, Brazil.; 6 Escola Paulista de Medicina da Universidade Federal de São Paulo (EPM-Unifesp), São Paulo, SP, Brazil.

**Keywords:** Fallopian tubes, Ovary/embryology, Organogenesis, Imaging, three-dimensional, Tomography, X-ray computed/methods, Tubas uterinas, Ovário/embriologia, Organogênese, Imagem tridimensional, Tomografia computadorizada/métodos

## Abstract

**Objective:**

To evaluate and reconstruct three-dimensional images of vascularization along the fallopian tube (FT), as well as to determine its relationship with the ovary and ovarian fimbria, and to quantify the blood vessels along the FT according to its anatomical segments, using confocal microtomography (micro-CT).

**Materials and Methods:**

Nine specimens (six FTs and three FTs with ovaries) were fixed in a solution of 10% formalin for > 24 h at room temperature. Iodine staining was performed by soaking the specimens in 10% Lugol’s solution for 24 h. All specimens were evaluated using micro-CT. A morphometric analysis was performed on the reconstructed images to quantify the vascular distribution along the FT.

**Results:**

In the FTs evaluated, the density of blood vessels was significantly greater in the fimbrial segments than in the isthmic segments (*p* < 0.05). The ovarian fimbria was clearly identified, demonstrating the important relationship between these vessels and the FT fimbriae.

**Conclusion:**

We believe that the vascularization in the fimbriae is greater than and disproportional that in the other segments of FT, and that the ovarian fimbria plays an important role in the development of that difference.

## Abstract (pt)

**Objetivo:**

Avaliar e reconstruir imagens tridimensionais da vascularização ao longo da trompa de Falópio (TF) e sua relação com o ovário e fímbria do ovário, além de quantificar os vasos sanguíneos ao longo da TF de acordo com seus segmentos anatômicos usando microtomografia confocal (micro-TC).

**Materiais e Métodos:**

Nove espécimes (seis com TFs e três com TFs e ovários) foram fixados em solução de formol a 10% por mais de 24 h à temperatura ambiente. A coloração com iodo foi realizada usando solução de Lugol a 10% durante 24 h. Todos os espécimes foram avaliados usando micro-TC. Uma análise morfométrica foi realizada nas imagens reconstruídas para quantificar a distribuição vascular ao longo da TF.

**Resultados:**

A densidade dos vasos sanguíneos nas fímbrias foi significativamente aumentada em comparação ao segmento ístmico da TF (*p* < 0,05). A fímbria ovariana foi claramente identificada, demonstrando importante relação entre esses vasos e as fímbrias da TF.

**Conclusão:**

Acreditamos que as fímbrias apresentam vascularização aumentada e desproporcional em comparação com os demais segmentos da TF e que a fímbria ovariana desempenha importante papel nessa diferença.

## INTRODUCTION

The human oviduct has been present in the medical literature for almost 3,000 years and was first described, by the Greek physician Soranus of Ephesus (circa AD 100), as the conduit linked to fertilization. In his masterpiece entitled “De Humani Corporis Fabrica”^(1)^, Andreas Vesa lius renamed it the fallopian tube (FT) after his assistant Gabriele Fallopio (1523-1562). However, in addition to the fundamental role that the FT plays in human fertilization, recent evidence suggests that most serous ovarian carcinomas (the most aggressive and deadly gynecological malignancies) originate from the distal FT rather than from the ovarian surface epithelium^(2-5)^. Consequently, the International Federation of Gynecology and Obstetrics staging classification for ovarian, tubal, and peritoneal cancers was revised in 2014^(6)^. That rekindled interest in FT physiology and pathology. There have been increasing numbers of studies involving the cellular and molecular aspects of the FT, mainly the mucosa in the fimbriae, the most common location for tubal malignant transformation^(7,8)^. Distinctive stem cell markers were recently identified in the fimbriae, suggesting that the fimbriae have a unique embryonic development, separate from other tubular segments^(9,10)^. However, information about vascularization along the FT is scarce. One report, by Stange^(11)^, described the vascular distribution along the FT as “disproportional”, increasing among the fimbriae.

In this study, we aimed to observe the distribution of blood vessels along the FT, focusing on the infundibulum and its relationship with the ovary. To that end, we employed confocal microtomography (micro-CT), a nondestructive X-ray technology that provides high-resolution three-dimensional images of *ex vivo* specimens.

## MATERIALS AND METHODS

### Patients

We included nine patients who were scheduled to undergo surgical procedures for the treatment of benign gynecological conditions, such as uterine adenomyosis and uterine fibroid, or surgical sterilization. The study was approved by the Research Ethics Committee of Vassouras University (Reference no. 56031916.0.0000.5290), in the municipality of Vassouras, Brazil, and all participating patients gave written informed consent. Patients who had a macroscopically abnormal FT or ovary were excluded, as were those suspected of having malignant conditions.

### Specimen preparation

Nine specimens (six FTs and three FTs with ovaries) were fixed in 10% formalin for > 24 h at room temperature^(12)^. After washing twice with distilled water, iodine staining was performed by soaking the specimens in 10% Lugol’s solution for 24 h, as previously described for biological samples^(13)^. The specimens were removed from the staining solution and rinsed with phosphate-buffered formalin to remove excess stain and prevent surface saturation. To hold the specimens in place, ensure the mechanical stability, and avoid movement artifacts during micro-CT, the specimens were fixed onto Styrofoam. After the micro-CT scan, the specimens were returned to 10% formalin to de-stain and to prepare for histological analysis.

### Micro-CT and images analysis

For the acquisition of FT images, we employed a micro-CT system (SkyScan 1173 v.1.6.9.4; Bruker microCT, Kontich, Belgium). The parameters (X-ray energy, current, and exposure time) were individually adapted to optimize the images for each specimen. The following parameters were used for image acquisition: energy, 40 kV; current, 200 µA; exposure time, 250-100 ms; scan duration, 24-77 min; and voxel size, 11.04-21.01 µm. Post-processing analysis and image reconstructions were performed using the software CTan, version 1.16 (Bruker microCT).

### Morphometric analysis-quantifying vessel density

To quantify the vascular distribution along the FT, a morphometric analysis was performed on images reconstructed using the software CTan. Images were classified into regions of interest: fimbriae, ampulla, and isthmus. Following classification, the FT lumen was excluded and the total volume of each segment was quantified. The quantitative measurement of vascular volume was calculated using the morphometric parameter of connectivity^(14)^. To remove the maximum number of artifacts and isolated radiopaque signals, continuous, connected radiopaque images of the vessels were selected. The total volume of the vessels within each segment was reported in voxels.

To quantify the vessel density within each region of the specimen, the ratio between the total FT volume and the FT blood volume was analyzed. Statistical analysis was performed using the Predictive Analytics Software package, version 18.0 for Windows (SPSS Inc., Chicago, IL, USA) and GraphPad Prism (GraphPad Software Inc., San Diego, CA, USA). We calculated the mean, standard deviation, median, 25th and 75th percentiles, as well as minimum and maximum values. Since the sample size was too small to properly evaluate the distribution, nonparametric tests were used. Friedman and Dunn’s tests were used in order to compare the different regions of the uterine tubes. Statistical significance was defined as *p* < 0.05.

## RESULTS

The vascular distribution of disease-free FTs from women of reproductive age was evaluated after patients underwent a total abdominal hysterectomy or salpingectomy. The uterus was separated from the FT because of the lack of morphological references and methods to calculate its vascularization. The easily recognizable FT mucosa was vital to selecting the FT segments.

**Figure 1:**
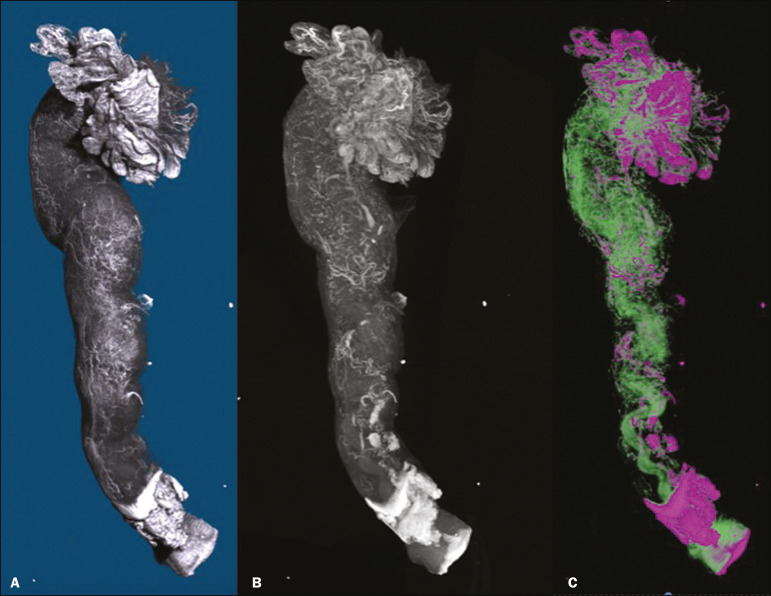
FT reconstructed on micro-CT and stained with iodine. **A:** Surface reconstruction. **B:** Three-dimensional CT reconstruction. Note the evident contrast of contiguous images. **C:** Reconstruction of the vessels along the FT (in pink). The tubal mucosa is separated in green.

The vascular distribution of the FT was visible in all the specimens. The specimens presented outstanding resolution and demonstrated a clear three-dimensional vessel network. Although the Lugol’s solution was readily absorbed into the larger vessels, very small vessels and capillaries could not be detected. The voxel size and inadequacies in connectivity limited our ability to study the smaller vessels. The vessel endothelium was also visible, with greater intensity than the muscle segments of the FT. The vessel endothelium was visible even in the vessels without blood (Figure 1).

Table 1 shows the descriptive statistics for total volume, vascular volume, and vascular proportion in the fimbrial, ampullary, and isthmic segments of the FT. Differences between the fimbrial and isthmic segments of the FT were significant (*p* < 0.05). The values of the fimbriae and ampulla were not statistically different, although they did demonstrate increasing vascularization toward the distal part of the FT (Table 2).

**Table 1:** Descriptive analysis of the sample.

Variable	Mean	Standard deviation	Median	25th-75th percentile	Minimum-Maximum
Total fimbriae volume (voxels)	248.7	237.9	176.0	97.1-374.1	54.4-713.1
Total ampulla volume (voxels)	269.4	182.7	270.8	91.3-378.6	85.7-586.2
Total isthmus volume (voxels)	87.79	72.60	80.33	25.32-138.20	7.79-214.30
Vascular fimbriae volume (voxels)	7.25	4.63	5.65	3.88-11.33	2.61-15.23
Vascular ampulla volume (voxels)	5.04	4.02	3.13	2.36-8.99	1.44-11.81
Vascular isthmus volume (voxels)	0.82	0.79	0.58	0.13-1.69	0.11-1.92
Vascular proportion in fimbriae (%)	4.17	3.04	3.18	1.97-6.48	1.44-9.68
Vascular proportion in ampulla (%)	2.22	1.34	2.44	0.78-3.29	0.55-3.98
Vascular proportion in isthmus (%)	1.08	0.84	0.93	0.39-1.69	0.34-2.46

**Table 2:** Comparisons between the different segments of the FT (n = 6).

Variable	Friedman test		Dunn’s multiple comparisons test	
		Fimbrial vs. ampullary	Fimbrial vs. isthmic	Ampullary vs. isthmic
Total volume	0.006	Non-significant	Non-significant	< 0.05
Vascular volume	0.006	Non-significant	< 0.05	Non-significant
Vascular proportion	0.01	Non-significant	< 0.05	Non-significant

**Figure 2:**
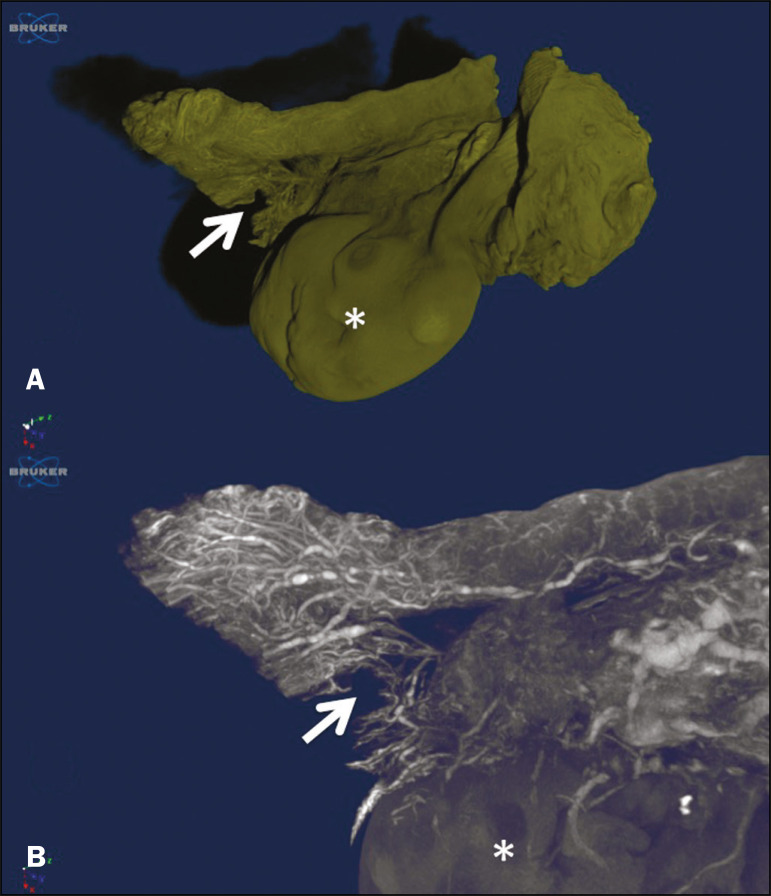
Left FT and ovary from a hysterectomy and salpingo-oophorectomy. **A:** Surface reconstruction of the adnexa, demonstrating part of the myometrium on the right. The ovary was normal (asterisk), and the arrow shows the ovarian fimbria. **B:** CT image of the left adnexa. Note the intense vascularization of the fimbriae compared with the other segments of the tube and the vascular connection from the ovary (asterisk) to the fimbriae (arrow, ovarian fimbria).

The presence of vessels in the ovarian fimbria was effortlessly visualized and reconstructed in three-dimensional imaging (Figure 2), demonstrating the important vascular relationship between the FT fimbriae and the ovarian fimbria. The characteristics of the ovarian fimbria, such as the number and thickness of vessels, were not evaluated.

## DISCUSSION

The present study aimed to observe the distribution of blood vessels along the FT. In addition to its function as a conduit between the uterus and ovary for gamete fertilization, the FT has been associated with many gynecological disorders. It is known to be the pathway for retrograde menstruation and development of ovarian endometrioid or clear cell carcinoma^(15)^, it is associated with the spread of tubal and ovarian high-grade serous carcinoma into the endometrium^(16)^, and it is frequently involved with ascending infections in pelvic inflammatory disease. Recent conceptualizations for the pathogenesis of serous ovarian tumors have focused on their possible origins within the FT^(5)^. After seminal sources suggested that the FT is a source of ovarian and peritoneal tumors, many researchers discovered a stem cell-like enrichment of the fimbriae and suggested that the fimbriae have a different developmental origin from the rest of the tube^(9)^. This was also previously described, in cases of isolated ovarian agenesis without the accompanying fimbriae but with an otherwise normal FT^(17,18)^. In the present study, we were able to demonstrate increased vascularization in the distal segments of FT. The absence of a statistically different fimbriae-ampulla ratio notwithstanding, the method employed was able to quantify variability in the vasculature and to emphasize the association between the ovarian fimbria and FT fimbriae. The ovarian fimbria establishes the ovarian surface epithelium-ligament-fimbriae epithelial continuum, which plays a recognized role in FT vascularization^(19)^. In our study, the ovarian fimbria demonstrated an intense, localized vascular connection with the FT fimbriae and is likely the driving force behind the increased vascularization in this segment. To our knowledge, Stange^(11)^ was the first to describe this disproportional, increased vascularization and considered the observation to be a result of the absence of a bursa ovarica and of fimbriae. Other studies have described various temperature gradients along the FT and ovary, which they have attributed to relative blood flow distribution, because sperm has the ability to be directionally guided through the oviduct by temperature changes^(20,21)^.

A number of recent studies conducted in Brazil have highlighted the importance of imaging methods in the evaluation of the female reproductive system^(22-27)^. The use of micro-CT was pivotal to observing the vascularization in detail. Over the past few years, since the application of contrast agents to increase radiographic contrast of small organic samples became common, impressive progress has been made in the study of small soft-tissue samples with micro-CT. Contrast varies depending on the tissue specificity of the contrast agent, as well as its cost, toxicity, and overall effect on the histologically prepared specimen. Iodine, which is the most frequently used contrast agent in micro-CT, has several advantages, such as low toxicity, low cost, rapid staining, and perceived non-destructiveness, allowing biological specimens to be analyzed and subsequently used in conventional microscopy^(13,28)^. In human tissues, the *ex vivo* use of micro-CT includes unenhanced studies-for intraoperative breast cancer^(29,30)^, lungs^(31)^, bones^(32)^, and dental pathologies^(33,34)^-and contrast-enhanced studies-for the brain^(35)^, fetal heart^(36)^, post-mortem lower digestive tract obstruction imaging^(37)^, and human autopsy^(38)^. Some methods used for phenotyping FTs have good histopathological correlation^(39,40)^; however, those methods are not able to reconstruct the FT vasculature microscopically.

Micro-CT allows the quantification of soft-tissue morphology, including linear and volumetric data, and has been used for many vascular reconstructions in animals^(41)^. In the present study, an “angiogram-like” image was produced due to the high affinity between iodine and blood. However, smaller vessels and vessels not containing blood may have been missed, the observed affinity between iodine and endothelium being the exception. The absence of small vessels in the continuum image precluded the use of an automated system to reconstruct the vessels. Further studies are needed in order to optimize fixation, the staining process, and image acquisition for gynecologic specimens. The present study was also limited by the small number of specimens and therefore could not measure the influence of patient age, hormone therapy, and the various surgical techniques performed during the specimen collection process.

In summary, we believe that the FT fimbrial segment has a disproportional and increased vascularization compared with the other segments and that the ovarian fimbria plays an important role in the development of those differences. Larger, multicenter studies are needed in order to confirm these findings, to determine the influences of the ovarian surface epithelium-ligament-fimbriae epithelial continuum, and to identify the roles that both play in tubal and ovarian physiology and pathologies.
